# Mathematics in Biomedical Imaging

**DOI:** 10.1155/2007/64954

**Published:** 2007-11-19

**Authors:** Ming Jiang, Alfred K. Louis, Didier Wolf, Hongkai Zhao, Christian Daul, Zhaotian Zhang, Tie Zhou

**Affiliations:** ^1^School of Mathematical Sciences, Peking University, Beijing 100871, China; ^2^Institute for Applied Mathematics, Saarland University, Postfach 151150, 66041 Saarbrücken, Germany; ^3^Institut National Polytechnique de Lorraine, Centre de Recherche en Automatique de Nancy, Vandoeuvre-Les-Nancy 54516, France; ^4^Department of Mathematics, University of California Irvine, Irvine, CA 92697, USA; ^5^Division of Electronics & Information System, Department of Information Science, National Natural Science Foundation of China, Beijing 100085, China

Biomedical imaging is critically important for life science and health care. In this rapidly developing field, mathematics is one of the most powerful tools for developing image reconstruction as well as image processing theory and methods. Many of the innovations in biomedical imaging are fundamentally related to the mathematical sciences. With improvements of traditional imaging systems and emergence of novel imaging modalities such as molecular imaging towards molecular medicine, imaging equations that link measurements to original images become increasingly more complex to reflect the reality upto an ever-improving accuracy. Mathematics becomes increasingly useful and leads to a new array of interdisciplinary and challenging research opportunities.The future biomedical imaging will include advanced mathematical methods as major features.

It is a current trend that more mathematicians become engaged in biomedical imaging at all levels, from image reconstruction to image processing, and upto image understanding and various high level applications. This special issue addresses the role of mathematics in biomedical imaging. The themes include theoretical analysis, algorithm design, system modeling and assessment, as well as various biomedical imaging applications. From 10 submissions, 7 papers are published in this special issue. Each paper was reviewed by at least two reviewers and revised according to review comments. The papers cover the following imaging modalities: X-ray computed tomography (CT), positron emission tomography (PET), magnetic resonance imaging (MRI), diffusion tensor imaging (DTI), electrical impedance tomography (EIT), and elasticity imaging using ultrasound.

The field of X-ray imaging has been expanding rapidly since Röntgen's historical discovery in 1895. X-ray CT, as the first noninvasive tomographic method, has revolutionized imaging technologies in general, which was also the first successful application of mathematics in biomedical imaging. The mathematics is the theory of Radon transform invented by Radon in 1917. Further research may rejuvenate this classic topic to meet modern imaging challenges such as scattering effects. In Truong et al.'s paper, the authors presented two further generalizations of the Radon transform, namely two classes of conical Radon transforms which originate from imaging processes using Compton scattered radiation. The first class, called C1-conical Radon transform, is related to an imaging principle with a collimated gamma camera whereas the second class, called C2-conical Radon transform, contains a special subclass which models the Compton camera imaging process. They demonstrated that the inversion of C2-conical Radon transform can be achieved under a special condition.

PET is currently a major imaging modality for clinical diagnostics and pharmacological research. The expectation maximization (EM) algorithm has been used in PET for years. In Chan et al.'s paper, the authors propose to combine the level set method with the EM algorithm for PET. The level set method, which was originally developed for capturing moving interfaces in multiphase physics, is used here to capture geometric information, for example, the anatomical structure. If another type of information is available, for example, CT or MRI images, it can be used as prior knowledge and can be incorporated into the formulation. The idea of combining geometric information with statistic methods is quite interesting and promising.

In Mueller-Bierl et al.'s paper, the authors investigated the magnetic field distribution and signal decay of high field strength functional MRI imaging. The static dipole model has been extended to a dynamic model to describe the sampling of phases of the individual protons moving in the inhomogeneous magnetic field. The dynamic Brownian motion process is implemented using a Monte Carlo method with different step parameters. Various factors for signal decay and artifacts formation are investigated. Results from different methods were compared.

Earlier work on total variation (TV) regularization for color (vector valued) images is naturally extended to DTI, which is composed of a symmetric positive definite (SPD) matrix at each pixel. In the last decade, a new magnetic resonance modality, DTI has caught a lot of interest. DTI can reveal anatomical structure information. In this special issue, there are two papers on this imaging technique. In Christiansen et al.'s paper, this type of tensor-valued images is denoised using TV regularization. Recently, partial differential equation- (PDE-) based image processing methods have been very successful in many applications due to its intrinsic geometric nature. TV regularization, which can effectively remove noise while keeping sharp features, is one of the most important techniques for PDE-based image processing methods. Although TV regularization is very natural for scalar (gray) images, there is no easy and natural way to extend to vector values (color) images. This paper proposes to use TV regularization to denoise DTI based on previous work on generalizing TV to vector value images. To maintain the SPD structure of the tensor, the authors propose to work on the LU factorization of the tensor rather than on the tensor itself. The results demonstrated the expected strength of TV regularization. Another paper on DTI is by Duan. The author proposes a semi-automatic thalamus and thalamus nuclei segmentation algorithm based on the mean-shift algorithm. The main advantages of the proposed method over methods based on K-means are its flexibility and adaptivity, since assumptions of Gaussian or a fixed number of clusters are not needed.

The EIT and elasticity imaging in the following two contributions are inverse problems of partial differential equations. In EIT, electric currents are applied to the boundary of an object and the induced surface voltages are measured. The measured voltage data are then used to reconstruct the internal conductivity distribution of the object. In Azzouz et al.'s paper, the authors establish two reconstruction methods for a new planar electrical impedance tomography device. This prototype allows noninvasive medical imaging techniques if only one side of a patient is accessible for electric measurements. The two reconstruction methods have different properties: one is a linearization type method that allows quantitative reconstructions; and the other one, that is, the factorization method, is a qualitative one, and is designed to detect anomalies within the body. Numerical results are also presented. In elasticity imaging, tissue motion in response to mechanical excitation is measured using modern imaging systems, and the estimated displacements are then used to reconstruct the spatial distribution of Young's modulus. In Aglyamov et al.'s paper, the authors propose a novel reconstruction technique for elastic properties of biological tissues from compressional ultrasound elastography. The technique assumes spherical or cylindrical symmetry so that strain equations can be simplified. The reconstruction is conducted with inverse problem computations for partial differential equations. The proposed method is applied to image liver hemangioma (spherical symmetry) and rat DVT (cylindrical symmetry). The reconstruction results are compared with traditional elastography images. This paper offers some interesting thoughts especially for some special clinical cases where elasticity properties are spherical symmetric.

These papers represent an exciting, insightful observation into the state of the art, as well as emerging future topics in this important interdisciplinary field. We hope that this special issue would attract a major attention of the peers.

